# Supplementary material for: Group-Based Pelvic Floor Telerehabilitation to Treat Urinary Incontinence in Older Women: A Feasibility Study
Source: Int J Environ Res Public Health. 2023 May 11;20(10):5791. doi: 10.3390/ijerph20105791 (PMC10218421; doi:10.3390/ijerph20105791)
Supplement: Supplementary file 1 [file ijerph-20-05791-s001.zip › ijerph-2257450-supplementary.pdf]

CONSORT checklist of information to include when reporting a pilot trial

Section/ topic and item #	Standard checklist item	Extension for pilot trials	Page # where item is reported
**Title and abstract**			
1a	Identification as a randomized trial in the title	Identification as a pilot or feasibility randomized trial in the title	Page 1
1b	Structured summary of trial design, methods, results, and conclusions (for specific guidance see CONSORT for abstracts)	Structured summary of pilot trial design, methods, results, and conclusions (for specific guidance see CONSORT abstract extension for pilot trials)	Page 1
**Introduction**			
Background and objectives:			
2a	Scientific background and explanation of rationale	Scientific background and explanation of rationale for future definitive trial, and reasons for randomized pilot trial	Pages 1-2
2b	Specific objectives or hypotheses	Specific objectives or research questions for pilot trial	Page 2
**Methods**			
Trial design:			
3a	Description of trial design (such as parallel, factorial) including allocation ratio	Description of pilot trial design (such as parallel, factorial) including allocation ratio	Page 2
3b	Important changes to methods after trial commencement (such as eligibility criteria), with reasons	Important changes to methods after pilot trial commencement (such as eligibility criteria), with reasons	N/A
Participants:			
4a	Eligibility criteria for participants		Pages 2-3
4b	Settings and locations where the data were collected		Pages 3-4
4c		How participants were identified and consented	Pages 2-3, Page 4 (Supplementary table S3)
Interventions:			
5	The interventions for each group with sufficient details to allow replication, including how and when they were actually administered		Page 3 (single-arm)
Outcomes:			
6a	Completely defined pre-specified primary and secondary outcome measures, including how and when they were assessed	Completely defined pre-specified assessments or measurements to address each pilot trial objective specified in 2b, including how and when they were assessed	Pages 3-4

6b	Any changes to trial outcomes after the trial commenced, with reasons	Any changes to pilot trial assessments or measurements after the pilot trial commenced, with reasons	N/A
6c		If applicable, pre-specified criteria used to judge whether, or how, to proceed with future definitive trial	N/A
Sample size:			
7a	How sample size was determined	Rationale for numbers in the pilot trial	Reference 40 (complete published protocol), cited page 2, page 3
7b	When applicable, explanation of any interim analyses and stopping guidelines		N/A
Randomization:			
Sequence generation:			
8a	Method used to generate the random allocation sequence		N/A
8b	Type of randomization; details of any restriction (such as blocking and block size)	Type of randomization(s); details of any restriction (such as blocking and block size)	N/A
Allocation concealment mechanism:			
9	Mechanism used to implement the random allocation sequence (such as sequentially numbered containers), describing any steps taken to conceal the sequence until interventions were assigned		N/A
Implementation:			
10	Who generated the random allocation sequence, enrolled participants, and assigned participants to interventions		N/A
Blinding:			
11a	If done, who was blinded after assignment to interventions (eg, participants, care providers, those assessing outcomes) and how		N/A
11b	If relevant, description of the similarity of interventions		N/A
Analytical methods:			
12a	Statistical methods used to compare groups for primary and secondary outcomes	Methods used to address each pilot trial objective whether qualitative or quantitative	Page 3-4

12b	Methods for additional analyses, such as subgroup analyses and adjusted analyses	Not applicable	N/A
**Results**			
Participant flow (a diagram is strongly recommended):			
13a	For each group, the numbers of participants who were randomly assigned, received intended treatment, and were analyzed for the primary outcome	For each group, the numbers of participants who were approached and/or assessed for eligibility, randomly assigned, received intended treatment, and were assessed for each objective	Page 4, Page 9 (Figure 1)
13b	For each group, losses and exclusions after randomization, together with reasons		Page 4, Page 9 (Figure 1)
Recruitment:			
14a	Dates defining the periods of recruitment and follow-up		Page 4
14b	Why the trial ended or was stopped	Why the pilot trial ended or was stopped	N/A
Baseline data:			
15	A table showing baseline demographic and clinical characteristics for each group		Page 5
Numbers analyzed:			
16	For each group, number of participants (denominator) included in each analysis and whether the analysis was by original assigned groups	For each objective, number of participants (denominator) included in each analysis. If relevant, these numbers should be by randomized group	Pages 4-7, Table 2
Outcomes and estimation:			
17a	For each primary and secondary outcome, results for each group, and the estimated effect size and its precision (such as 95% confidence interval)	For each objective, results including expressions of uncertainty (such as 95% confidence interval) for any estimates. If relevant, these results should be by randomized group	N/A (no comparisons)
17b	For binary outcomes, presentation of both absolute and relative effect sizes is recommended	Not applicable	N/A
Ancillary analyses:			
18	Results of any other analyses performed, including subgroup analyses and adjusted analyses, distinguishing pre-specified from exploratory	Results of any other analyses performed that could be used to inform the future definitive trial	N/A
Harms:			
19	All important harms or unintended effects in each group (for specific guidance see CONSORT for harms)		Page 6, Table 2

19a		If relevant, other important unintended consequences	N/A
**Discussion**			
Limitations:			
20	Trial limitations, addressing sources of potential bias, imprecision, and, if relevant, multiplicity of analyses	Pilot trial limitations, addressing sources of potential bias and remaining uncertainty about feasibility	Page 12
Generalizability:			
21	Generalizability (external validity, applicability) of the trial findings	Generalizability (applicability) of pilot trial methods and findings to future definitive trial and other studies	Page 12
Interpretation:			
22	Interpretation consistent with results, balancing benefits and harms, and considering other relevant evidence	Interpretation consistent with pilot trial objectives and findings, balancing potential benefits and harms, and considering other relevant evidence	Pages 10-12
22a		Implications for progression from pilot to future definitive trial, including any proposed amendments	Page 12
**Other information**			
Registration:			
23	Registration number and name of trial registry	Registration number for pilot trial and name of trial registry	Page 2
Protocol:			
24	Where the full trial protocol can be accessed, if available	Where the pilot trial protocol can be accessed, if available	Reference 40 (complete published protocol), cited page 2, page 3
Funding:			
25	Sources of funding and other support (such as supply of drugs), role of funders		Page 12
26		Ethical approval or approval by research review committee, confirmed with reference number	Page 12

Supplementary Figure S1. Weekly program delivery fidelity for the four main pelvic floor exercises over the 12-week program duration. (**A**) Maximal pelvic floor contraction exercise; (**B**) Knack/Coughing exercise; (**C**) Fast contractions exercise; (**D**) Podium exercise.

A.

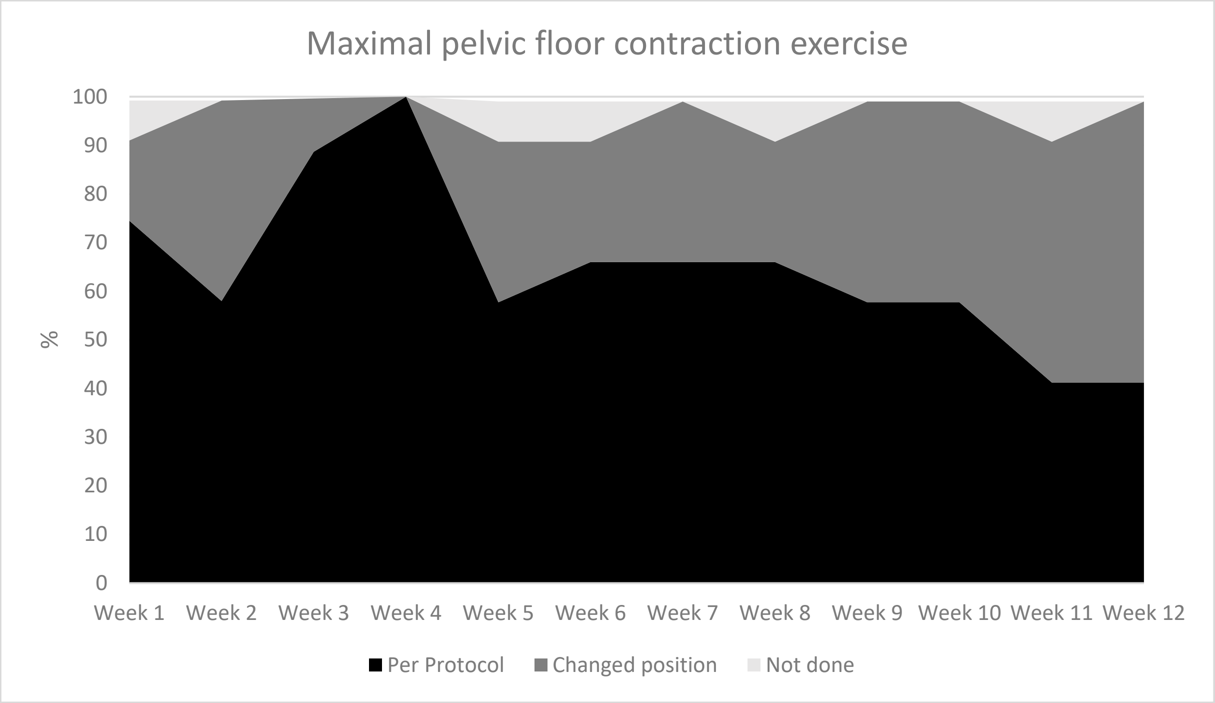

B.

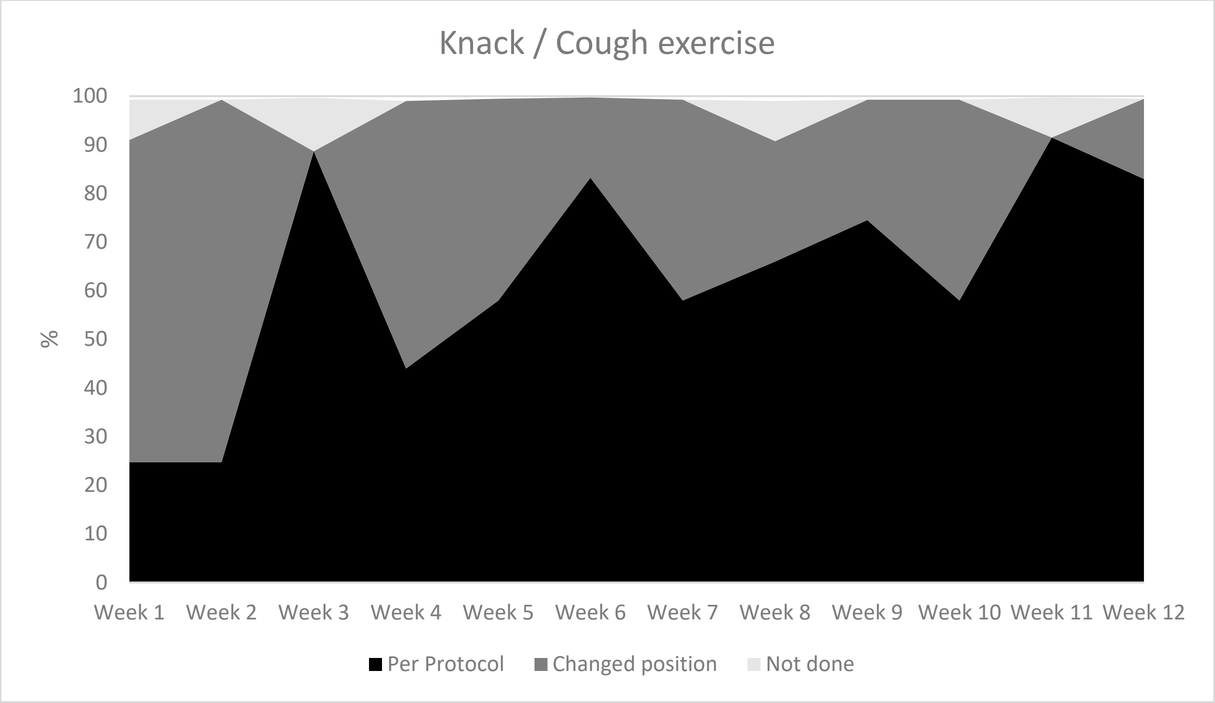

C.

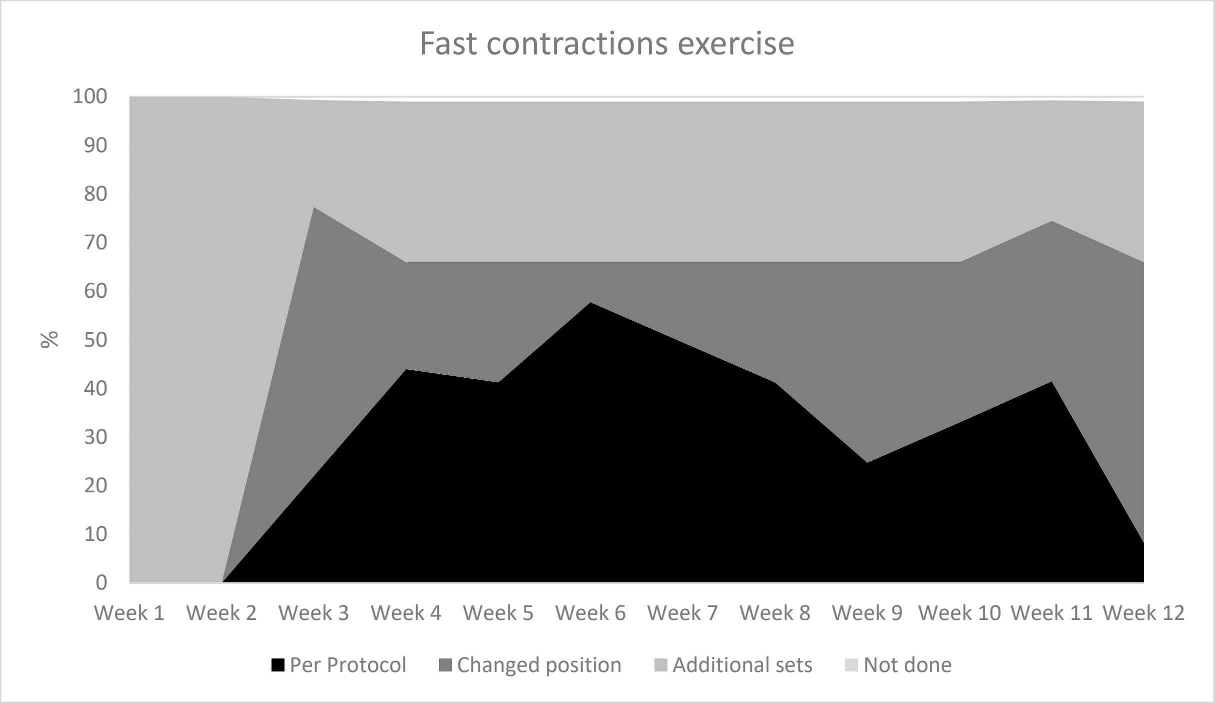
Fast contractions exercise
100
90
80
70
60
50
40
30
20
10
0
%
Week 1
Week 2
Week 3
Week 4
Week 5
Week 6
Week 7
Week 8
Week 9
Week 10
Week 11
Week 12
Per Protocol
Changed position
Additional sets
Not done

D.

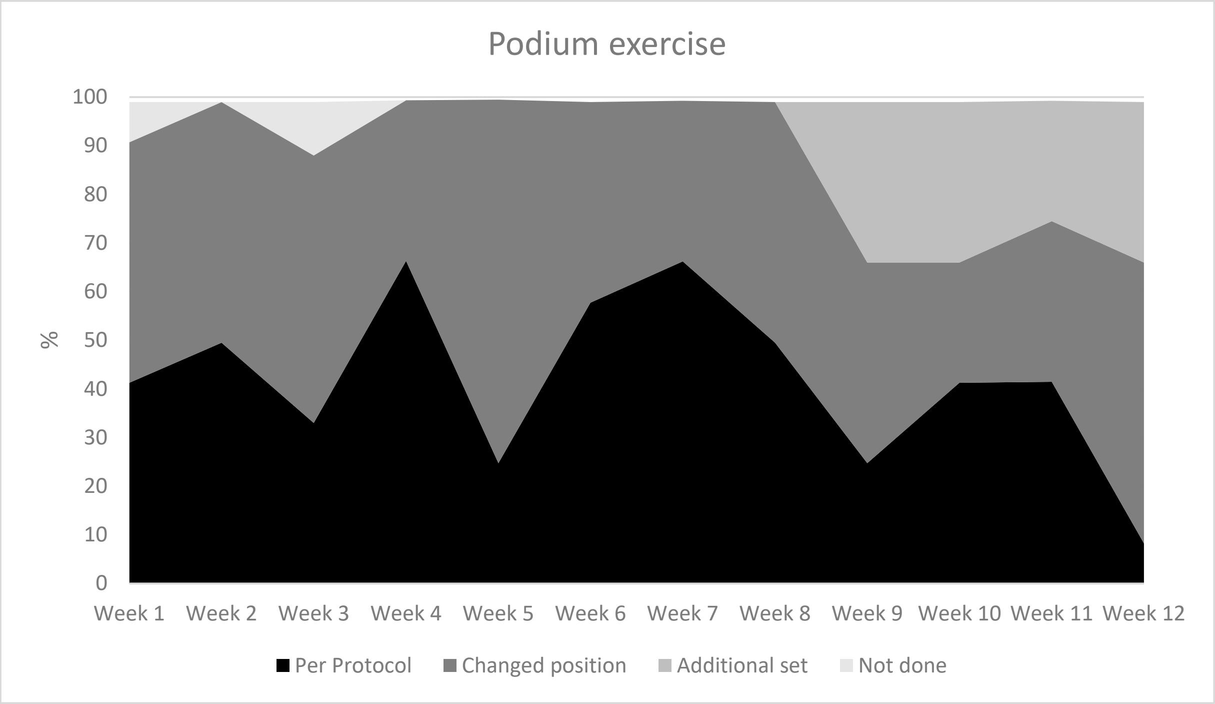
Podium exercise
100
90
80
70
60
50
40
30
20
10
0
%
Week 1
Week 2
Week 3
Week 4
Week 5
Week 6
Week 7
Week 8
Week 9
Week 10
Week 11
Week 12
Per Protocol
Changed position
Additional set
Not done

Supplementary Table S1. Detailed description of the online group-based pelvic floor muscle training program.

Item	Description	Online group-based pelvic floor muscle training
**1**	Detailed description of exercise equipment	To demonstrate the exercises, the physiotherapist had a floor mat and chair. To host the online group-based program, the physiotherapist also had a laptop or desktop computer with internet access, a microphone and camera. The physiotherapist's device was equipped with Zoom 5.10 software (pro license) and a modified version of Step Mania 5.0.12 software.<br><br>The participants were encouraged to use a floor mat and chair to perform the exercises. They also needed a smartphone, tablet, laptop or desktop computer with internet access. They were offered support to install the Zoom 5.10 software (free version) if interested or could join directly from their internet browser.
**2**	Detailed description of instructor expertise, qualifications and/or training	Expertise: physiotherapist specialized in pelvic floor rehabilitation.<br><br>Qualification: postgraduate program in pelvic floor rehabilitation at Université de Montréal.<br><br>Program training: 4-h workshop and written treatment guidelines with a checklist.
**3**	Describe whether exercises were performed individually or in a group	Exercises were performed in a group of 6-8 women.
**4**	Describe whether exercises were supervised or unsupervised; how they were delivered	A trained physiotherapist led the sessions and supervised the exercises.
**5**	Detailed description of how adherence to exercise was measured and reported	Attendance to weekly sessions: The physiotherapist recorded the adherence to each supervised weekly session of the online group-based program using an attendance sheet.<br><br>Adherence to home exercises: During the 12-week program, participants filled an exercise diary and reported their numbers to the physiotherapist every week during their individual exchange period. Exercise diaries included the number of completed contractions per set, sets per day and days per week for each of the 4 exercises in the program (strength, speed of contraction, endurance and coordination).
**6**	Detailed description of motivation strategies	Motivational prompts were included in the exercise diaries.<br><br>At the beginning of each weekly session, women reported their adherence to home exercise. The physiotherapist provided tips and advice to improve adherence.<br><br>One education session (10-15 minutes) was dedicated to motivation, exercise adherence, exercise maintenance and strategies to adopt in case of relapse.
**7a**	Detailed description of decision rule(s) for determining exercise progression	The progression of the online exercise sessions and home exercises was standardized.

**7b**	Detailed description of how exercise program was progressed	The 12-week program included three phases of gradual exercise progression, lasting four weeks each. The phases comprised the gradual addition of increasingly difficult exercises in terms of duration, repetition, and body position.
**8**	Detailed description of each exercise to enable replication	See item 13.
**9**	Detailed description of any home program component	Four pelvic floor muscles exercises (same as in the weekly physiotherapy program): maximal contraction (strength), fast contractions (speed of contraction), podiums (endurance) and knack maneuver (coordination).<br><br>The home exercise program component was performed five days per week for the duration of the 12-week program.<br>The home program followed the treatment protocol progression. It was divided into three phases, allowing for gradual exercise progression; that is, the gradual addition of increasingly difficult exercises in terms of duration, repetition and position. Each of the three phases lasted four weeks.
**10**	Describe whether there were any non-exercise components	A 10-15-minute educational component was presented at every weekly session covering the anatomy and various functions of the pelvic floor muscles; normal bladder control; voiding parameters; bladder irritants; fluid intake; toilet positions; and voiding dynamics. Details of the content of each weekly session can be found in the previously published protocol [32].
**11**	Describe the type and number of adverse events that occurred during exercise	The type and number of adverse events are reported in the results section and Table 2 of the article.
**12**	Describe the setting in which the exercises were performed	The physiotherapist hosted the weekly sessions either from a treatment room at the research center in Montreal, their personal office or from their home.<br><br>Participants attended the weekly sessions remotely from their home or from where they were currently living at that time (they continued their participation when they were away on vacation, away for work, or when visiting their family).
**13**	Detailed description of the exercise intervention	After the 1-3-minute individual exchange period and the 10-15-minute educational component, the program included a 30-45-minute pelvic floor muscle training exercise component. The details of the exercises, contraction duration, and number of sets and repetitions can be found in the previously published guideline of the original in-person program [15].
**14a**	Describe whether the exercises were generic (one size fits all) or tailored	Generic, with some suggested adaptations for the positions in case of joint pain or difficulty.
**14b**	Detailed description of how exercises were tailored to the individual	Other than the adaptation for body positions in case of pain, the overall exercises were not tailored to the individual.
**15**	Describe the decision rule for determining the starting level	The starting level was standardized. All participants started at the same level, which progressed every four weeks.<br><br>If the participant had difficulty completing the program, she was encouraged to do as much as possible every time.
**16a**	Describe how adherence or fidelity to the intervention was assessed/measured	The fidelity to the intervention was assessed using the written treatment protocol conformity checklist. A physiotherapy doctoral student observed four sessions and independently completed the same conformity checklist to ensure the validity of the self-report conformity checklist answers.

16b	Describe the extent to which the intervention was delivered as planned	The extent to which the intervention was delivered as planned is reported in the study results section and Table 4 of the article.

Supplementary Table S2. Promotion strategies and recruitment

Promotion strategy	Women contacted N, (%)	Eligible participants N (%)
Community groups, associations or public events	51 (34%)	15 (44%)
Journals or magazines	32 (21%)	8 (24%)
Participant database	22 (15%)	2 (6%)
Word of mouth and past experiences with the research team	16 (11%)	6 (18%)
Social media	7 (5%)	1 (3%)
Collaborating clinics and healthcare professionals	6 (4%)	0 (0%)
Television	6 (4%)	0 (0%)
Radio	5 (3%)	2 (6%)
Did not remember or unable to reach and no information available	5 (3%)	0 (0%)
Total	150	34
